# The computational fluid dynamics-based epidemic model and the pandemic scenarios

**DOI:** 10.1063/5.0082090

**Published:** 2022-02-02

**Authors:** Talib Dbouk, Dimitris Drikakis

**Affiliations:** 1 IMT Nord Europe, Institut Mines-Télécom, University of Lille, F-59000 Lille, France; 2 University of Nicosia, Nicosia CY-2417, Cyprus

## Abstract

This study presents a computational fluid dynamics, susceptible–infected–recovered-based epidemic model that relates weather conditions to airborne virus transmission dynamics. The model considers the relationship between weather seasonality, airborne virus transmission, and pandemic outbreaks. We examine multiple scenarios of the COVID-19 fifth wave in London, United Kingdom, showing the potential peak and the period occurring. The study also shows the importance of fluid dynamics and computational modeling in developing more advanced epidemiological models in the future.

## I. INTRODUCTION

The Coronavirus (CoV) COVID-19^1^ is one of the most significant crisis events in modern history. It has been emerging as a global health and economic crisis worldwide. Governmental and political organizations have been facing considerable challenges in managing this crisis and its consequences, e.g., general lockdowns, hospitalizations, social distancing, and impact on the economy.

In the last 18 months, authors have presented several studies on multi-physics computational fluid dynamics (CFD) modeling and simulations to investigate the phenomena of airborne virus transmission for different conditions, such as coughing and social distancing, environmental effects, transmission in confined spaces, face masks, and pollen grain.^2–9^ In addition, several other authors have published papers in the Physics of Fluids, Special Collection “Flow and the Virus,”^10^ see, e.g., Refs. 11–15.

Previous studies emphasized^16^ the effectiveness of simple models when applied to the COVID-19 pandemic, such as the susceptible–infected–recovered (SIR) model.^17^ However, the SIR model contains only two parameters: a transmission rate (*β*) and a recovery rate (*γ*), which represent the probability per unit time that a susceptible individual becomes infected and the probability per unit time that an infected person becomes recovered and immunized, respectively. Nevertheless, scientists have been employing the SIR and the SIR-derived models to predict pandemic curves and epidemic outbreaks in many types of disease propagation.^18^

Dbouk and Drikakis^7^ coupled fluid dynamics and heat transfer with epidemiological prediction modeling. They developed a CFD-SIR-based model that can predict the epidemiological dynamics depending on the weather conditions such as the temperature, relative humidity (RH), and wind speed. In the CFD-SIR-based model, the concentration $C=C_{\mathrm{CoV}}$ of CoV particles in contaminated saliva droplets (suspended in the air), and the concentration rate (*CR*) as a function of *T*, *U_wind_*, and *RH* were computed. A new weather-dependent airborne infection rate ( AIR=β) index (*AIR* = *CR*) was introduced to indicate the viability of the airborne virus transmission as a function of *T*, *RH*, and *U*. Thus, AIR represents a weather-dependent transmission rate (physics-based) parameter.

The authors showed that taking the weather effects into account in epidemiological prediction models, such as the SIR model,^7^ can predict multiple annual pandemic outbreaks (waves). The current situation also confirms this as many countries face another pandemic wave. This is manifested by an increasing number of daily infections that challenge worldwide health systems. We believe that the fifth wave would occur regardless of the new Omicron variant or other mutations that might naturally arise. More transmissible variants would exaggerate the effects of the fifth or further waves.

Given the above, this study tends to shed light on the importance of

• employing weather-dependent pandemic predicting models;
• quantifying the effect of weather seasonality on the pandemic outbreak curves evolution; and
• social distancing in managing the pandemic's further waves due to seasonality factors.

## II. WEATHER-DEPENDENT CFD-SIR-BASED MODEL

We employed multi-phase CFD to investigate the Coronavirus concentration *C_CoV_* variation with time for a wide range of climate conditions [ 0≤T≤40 °C, (10≤RH≤90 %) and 4≤U≤20 km/h]. Studying several CFD simulation results, we developed and presented, for the first time, a reduced-order model (ROM) as an innovative virus airborne infection rate (AIR). The AIR index that is directly proportional to the virus concentration rate (CR) such that CR = AIR = *β*, detailed in Dbouk and Drikakis 2021.^7^

**FIG. 1.**
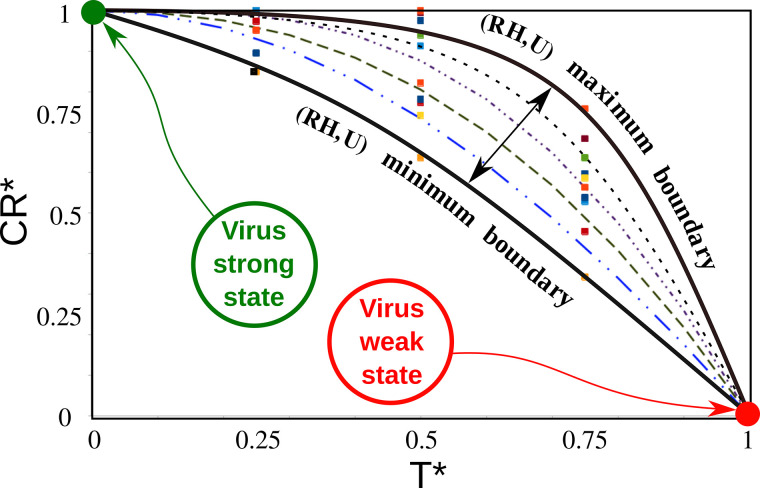
Scaling of the virus concentration rate (CR) with temperature (*T*), relative humidity (*RH*), and wind speed (*U*). Adapted from Dbouk and Drikakis, Phys. Fluids **33**, 021901 (2021). Copyright 2021 AIP Publishing.^7^

Figure 1 shows the scaling of the virus concentration rate (CR) with temperature (*T*), relative humidity (*RH*), and wind speed (*U*) modeled by

$$CR^{*}=F\;(RH^{*}+U^{*})\;\;{\text{sin}}^{*}(T^{*})+{\text{cos}}^{*}(T^{*}),$$ (1)

where F is given by

$$F=0.125\left(1-{\left(2T^{*}-1\right)}^{2}\right).$$ (2)

In Fig. 1, we observe that at high temperatures and low virus concentration rates, the virus is in a weak state, while at low temperatures and high virus concentration rates, the virus is in a strong state.

The AIR index, a new indicator for airborne virus transmission, is then introduced as a flow physics-relevant parameter in the epidemiological SIR model^17^ given by

$$\frac{dS}{dt}=-\beta \;S\;I/N,$$ (3)

$$\frac{dI}{dt}=\beta \;S\;I/N-\gamma I,$$ (4)

$$\frac{dR}{dt}=\gamma \;I.$$ (5)

β=AIR is a physics-based weather-dependent parameter, and *γ* is the recovery rate coefficient (depends mainly on a person's health and immunity system), *t* is time, and *N* is the total population number. *S*, *I*, and *R* are, respectively, the number of *susceptible*, *infected,* and *recovered* individuals. In other words, *β* is the probability per unit time that a susceptible individual becomes infected, and *γ* is the probability per unit time that an infected person becomes recovered and immune.

### A. Fifth wave pandemic predictions for London

**FIG. 2.**
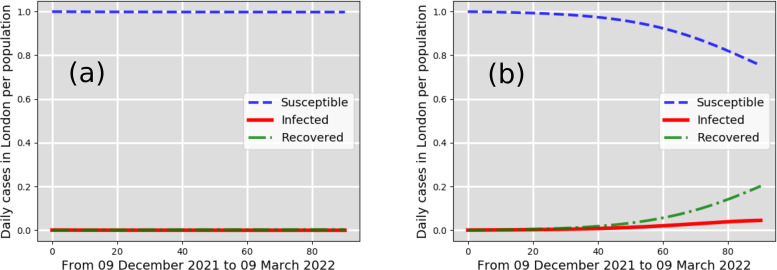
New cases per total population in the city of London, UK: infected (solid line), susceptible (dashed line), recovered (dashed-dotted line). The predictions consider as a starting point the 7761 infected individuals reported in London on 9 December 2021. Best-case scenarios (a) and (b) predicted by the CFD-SIR-based model are for $\beta =0.1\;{\text{days}}^{-1}$ and $\beta =0.2\;{\text{days}}^{-1}$, respectively.

**FIG. 3.**
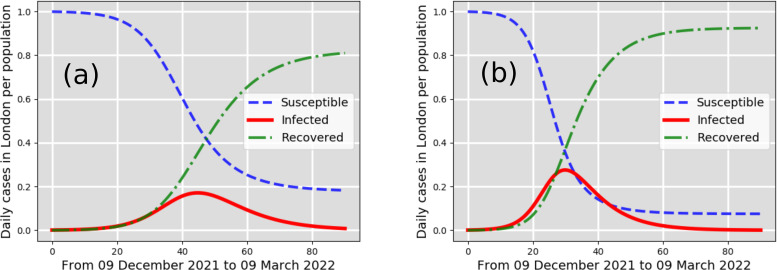
New cases per total population in London, UK: infected (solid line); susceptible (dashed line); recovered (dashed-dotted line). Starting from 7761 infected individuals reported in London on 9 December 2021. Moderate-case scenarios (a) and (b) predicted by the CFD-SIR-based model are for $\beta =0.3\;{\text{days}}^{-1}$ and $\beta =0.4\;{\text{days}}^{-1}$, respectively.

**FIG. 4.**
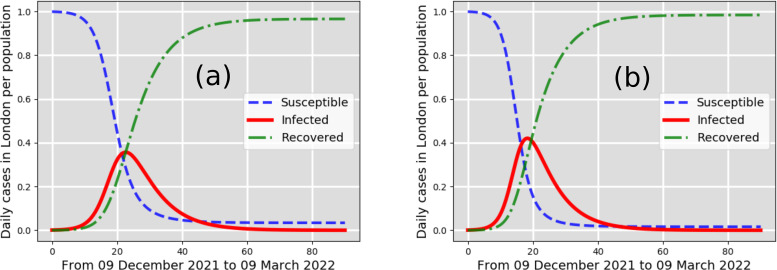
New cases per total population in London, UK: infected (solid line); susceptible (dashed line); recovered (dashed-dotted line). Starting from 7761 infected individuals reported in London on 9 December 2021. Worst-case scenarios, (a) and (b), predicted by the CFD-SIR-based model are for $\beta =0.5\;{\text{days}}^{-1}$ and $\beta =0.6\;{\text{days}}^{-1}$, respectively.

**FIG. 5.**
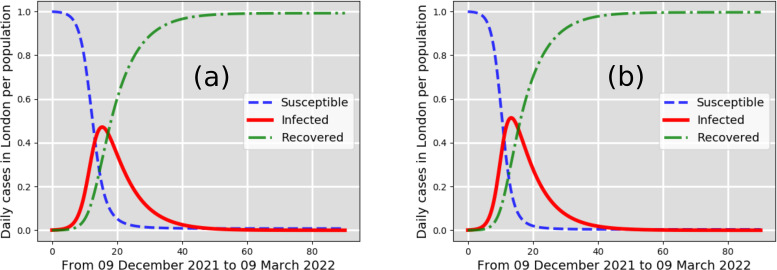
New cases per total population in London, UK: infected (solid line); susceptible (dashed line); and recovered (dashed-dotted line). Starting from 7761 infected individuals reported in London on 9 December 2021. Worst case scenarios (a) and (b) predicted by the CFD-SIR-based model are for $\beta =0.7\;{\text{days}}^{-1}$ and $\beta =0.8\;{\text{days}}^{-1}$, respectively.

From daily weather-data predictions of the temperature, relative humidity, and wind speed and employing the CFD-SIR-based model over three months from 9 December 2021 to 9 March 2022, we computed the pandemic curve for the daily number of cases in the city of London per total population. Starting from 9 December 2021 with 7761 infected cases reported in London,^19^ we predict the evolution of the pandemic curve for eight different scenarios in Figs. 2–5 [Fig. 2(a) is the best-case scenario; Fig. 5(b) is the worst-case scenario].

Figure 2 shows the pandemic curve for the best-case scenarios a and b. These two scenarios reveal that the total number of cases predicted between 9 December 2021 and 9 March 2022 does not exceed 4% of the total population of London. These two best-case scenarios, a and b, are a result of $\beta =0.1\;{\text{days}}^{-1}$ and $\beta =0.2\;{\text{days}}^{-1}$, respectively, which indicate a low airborne transmission rate. A low transmission rate in a winter season with cold, windy climate conditions, without strict lockdowns, can thus be only explained through the strict implementation of social measures and vaccinations. Considering reasonable social measures, including social distancing and face mask-wearing, one can observe from Fig. 3 that the infected cases in London can increase up to 19% and 23% of the total population (estimated to be around 8.982 × 10^6^ in the year 2019). The scenario of Fig. 3(a) shows that the infected cases could increase up to 19% in the pandemic curve (infected individuals) that might occur around 24 January 2022. The scenario of Fig. 3(b) also shows a peak in the pandemic curve (infected individuals) that might occur around 9 January 2022.

As worst-case scenarios represented by noncompliance to social distancing in London, without lockdowns, the pandemic curve predictions in Figs. 4 and 5 are steep. The peaks indicate that about 38% (around 1 January 2022) and about 42% (around 27 December 2021) of the population of London will be infected. The worst-case scenario [Fig. 5(b)] predicts that about 45% (around the Christmas Eve of 2021) of the population of London will be infected.

## III. CONCLUSIONS

We implemented the recently developed CFD-SIR-based epidemic model to predict the COVID-19 fifth wave in London, UK. The quantitative predictions for the three months: from 9 December 2021 to 9 March 2022, are presented and discussed for eight different scenarios. In a moderate scenario, we show that the infected cases could increase up to 19% in the pandemic curve (infected individuals) around 24 January 2022. The worst-case scenario predicts 38%, around 1 January 2022, and 42%, around 27 December 2021, infected.

Although some of the present results include periods before the publication of this study, we believe that the predictions of the pandemic curve, which depend on the weather conditions, could guide public authorities to better decide the social measures and future strategies. This is extremely important to prevent the spread of the virus and reduce hospitalizations. We conclude that

• employing weather-dependent pandemic prediction models is essential to precisely capture the behavior of multi-wave pandemic outbreaks annually;
• the predictions of the pandemic curves under several scenarios is important as it can guide the public authorities regarding social and financial strategies; and
• social distancing would continue to play a role in managing the pandemic outbreaks associated with more transmissible virus variants and seasonality.

## Data Availability

The data that support the findings of this study are available from the corresponding author upon reasonable request.
